# Vulvovaginal atrophy (VVA) in breast cancer survivors (BCS) is still an unmet medical need: results of an Italian Delphi Panel

**DOI:** 10.1007/s00520-019-05272-4

**Published:** 2020-01-22

**Authors:** Nicoletta Biglia, Lino Del Pup, Riccardo Masetti, Paola Villa, Rossella E. Nappi

**Affiliations:** 1 SCDU Ginecologia e Ostetricia, Ospedale Mauriziano Umberto I, Università di Torino, Torino, Italy; 2 SOC Ginecologia Oncologica, Centro di Riferimento Oncologico di Aviano (CRO) IRCCS, Aviano, Italy; 3 UOC Chirurgia Senologica, Policlinico Gemelli, Università Cattolica del Sacro Cuore, Rome, Italy; 4 Department of Obstetrics and Gynaecology, Fondazione Policlinico Universitario A. Gemelli IRCCS, Università Cattolica del Sacro Cuore, Rome, Italy; 5 UOSD Ostetricia e Ginecologia – Procreazione Medicalmente Assistita, IRCCS Fondazione Policlinico “S. Matteo”, Università degli Studi di Pavia, Pavia, Italy

**Keywords:** Vulvovaginal atrophy, Breast Cancer, Delphi Panel, Comorbidity, Burden of illness, Ospemifene

## Abstract

**Abstract:**

**Purpose:**

VVA is a common disease, with approximately 50% of all postmenopausal women having related symptoms. VVA has a significant impact on the personal and sexual lives and on many aspects of women’s self-esteem and emotional well-being. It is particularly frequent and severe in patients treated for BC, where it originates significant economic and social costs.

Given the lack of published evidence on this subject, a Delphi Panel was carried out to evaluate:

- The epidemiology of VVA and of its risk-factors/comorbidities in Italy
- The present standard of care and unmet medical needs
- The comparison between recent US epidemiological data and the Italian situation
- The health resources used in VVA BC

The burden of illnessDespite the considerable negative impact on quality of life, a disparity between the high prevalence of this condition and the infrequent clinical diagnosis is documented in medical practice and in surveys. This inaccuracy is thought to be primarily a consequence of patients’ unwillingness and/or reluctance to report symptoms in the clinical setting and of health-care professional’s difficulty in approaching this sensitive topic during routine consultations.

**Methods:**

A Delphi Panel methodology was used: a first round of written questionnaires, followed by a plenary meeting with a facilitator and by two additional rounds of telephone interviews.

**Results:**

The prevalence of the condition in Italy can be estimated in 115,000 cases out of 380,000 BC survivors.

The Panel confirmed that the epidemiological findings of a recent pharmacoeconomic analysis of a US claims database can be applied to Italian patient population.

The Panel confirmed also an estimate of 4.25 additional cases/100/yr of UTI (urinary tract infection) in VVA BC patients (vs. a non-VVA-matched population), of 3.68 additional cases of vulvovaginitis, of 6.97 cases of climacteric symptoms, and of 3.64 cases of bone and joint disorders.

As far as the resource use is concerned, in the VVA BC populations, 33.4 additional gynecological visits/100/year can be expected, along with 22.8 additional cancer screenings, 7.07 additional outpatient visits and 5.04 screenings for HPV.

**Conclusions:**

Even in Italy, a diagnosis of VVA, especially in a BC population, is associated with a relevant increase in the burden of illness and social costs, compared to a control population matched for age without VVA. This is due essentially to an increase in comorbidities and resource utilization with the consequence that an adequate treatment could reduce the impact of the condition.

## Purpose

Vulvar and vaginal atrophy (VVA) is a relatively common condition symptomatically affecting approximately 50% of all postmenopausal women [1].

The earliest symptoms of VVA are decreased vaginal lubrication, followed by other vaginal and urinary symptoms that may be exacerbated by superimposed infection, such as burning, itching, bleeding, leucorrhea, dyspareunia, and dysuria. These symptoms usually appear within 4–5 years after menopause [2–5].

Research has shown that the impact of VVA symptoms in postmenopausal women is significant. The scale of the problem is growing as a result of greater longevity, leading to a significant number of women spending more than one third of their life in the postmenopausal state.

The disparity between the high prevalence of this condition and the infrequent clinical diagnosis is documented in medical practice and in surveys despite the considerable negative impact on quality of life. This inaccuracy is thought to be primarily a consequence of patients’ unwillingness and/or reluctance to report symptoms in the clinical setting and health-care professional’s (HCP) difficulty of approaching this sensitive topic during routine consultations. The result of this underdiagnosis is a chronic and progressive condition that may not be addressed for long time and therefore more likely to undergo disease progression when left untreated [6].

During the last decade, many cross-cultural studies, predominantly surveys, have been conducted on postmenopausal women to gain new insight into the impact of VVA in current postmenopausal populations from different geographic distributions. These studies indicated that VVA symptoms have a global negative effect on sexual health, satisfaction, and sexual behavior, besides placing a relevant strain also on partners’ relationships [7]. Moreover, some surveys have clearly brought to light the lack of women’s knowledge in this area [6].

VVA symptoms are associated with decrements in quality of life that may be comparable to serious conditions such as arthritis, chronic obstructive pulmonary disease, asthma, and irritable bowel syndrome [1, 8].

These symptoms also feature prominently in women suffering from premature iatrogenic menopause that is often caused by cancer treatments. [9]. Moreover, local estrogen therapy, which is considered the standard of care for VVA symptoms, is contraindicated for some cancer survivors, leaving patients with a limited number of options [9, 10]. Approximately 50–75% of breast cancer survivors suffer from one or more VVA symptoms.

Aim of this research work, based on a Delphi Panel approach, was to assess the situation of VVA treatment in Italy, with particular emphasis of the situation of patients with a history of breast cancer (BC).

The Panel had also the objective to collect information on the experience of participants with new treatments for VVA (such as ospemifene) and on how these treatments are perceived in terms of their capability to reduce symptoms associated to VVA.

## Methods

The Panel was organized and run according to the “Delphi Panel” approach [11].

A questionnaire was prepared and circulated by e-mail to all participants, selected among the most relevant sites in Italy, in the second half of November 2016. The complete questionnaire is available upon request.

The responses were collected and analyzed in the days preceding the session, which took place in Milano on November 24, 2016. The results of the analysis of the questionnaires were summarized in a series of tables and slides that were presented during the sessions as a starting point for the discussion.

Two additional rounds of follow-up interviews took place in January 2017 and in July 2017. The final analyses were completed in December 2018.

All the members of the Panel participated in the preparation of the present work and are listed as authors.

## Results

The responses given by sites, integrated by a follow-up survey conducted during the months of December 2016 and January 2017, are detailed in Table 1.

**Table 1:** Approximated patient populations followed at the participating sites (figures taken from patient records)

	Site A	Site B	Site D	Site E
Number of pats followed per year	3000	2500	1500	1200
With VVA	700 (23%)	1250 (50%)	750 (50%)	500 (40%)
With VVA and BC	70 (10%)	35 (3%)	110 (15%)	250 (50%)
Pats treated with ospemifene	50	60	30	50

The centers, considered together, covered the vast majority of Italian regions, except for three areas (Valle D’Aosta, Trentino, and Liguria).

According to the members, VVA is a widely diffused and often underdiagnosed condition in Italy, with an estimated number of over 115,000 BC survivors, sexually active and with VVA [12].

This number, however, may increase in the future, due to the present trend toward extending adjuvant treatment in high-risk patients to more than 5 years.

The responses confirm that in patients with VVA without BC, the mainstays of treatment are lubricants and systemic and topical estrogens, while ospemifene is beginning to have an important role.

The therapeutic options are considerably reduced in patients with a history of BC, in whom hormone replacement therapy cannot be used.

Three sites reported the use of topical estrogens also in BC survivors, based on the indications of recent guidelines that would allow low dose topical estrogens also in patients with a history of BC unresponsive to lubricants and moisturizers, provided that they are not in treatment with aromatase inhibitors [13, 14].

Laser, whose efficacy has been very recently confirmed in the general population of VVA patients by the VeLVET trial [15], is viewed favorably by all Delphi Panel members but, in their practices, it is seldom used for cost reasons.

The long-term efficacy and safety of this treatment, moreover, has not yet been sufficiently demonstrated [16].

The responses and the following discussion confirm that VVA is accompanied by an additional relevant burden of comorbidities and resource use, both in patients with and without a history of BC.

During the discussion, the Panel reconsidered the frequency of the condition of “postmenopausal bleeding,” confirming that there is a higher frequency of events in the VVA population, especially in conjunction with sexual intercourse. According to the members, this unexpected bleeding is a cause of serious concern for patients and often originates visits to the Emergency Room and a series of clinical tests/assessments, to exclude the presence of an underlying neoplasm.

This subject was further elaborated during the two “follow-up” interviews where the Panel members agreed on the series of diagnostic and therapeutic procedures recommended after an episode of bleeding.

According to the Delphi Panel members, patients with a history of BC tend to suffer to a greater extent from the following conditions, some of which are related to VVA:

- Reduced quality of life
- Difficulties in sex life
- Depression and psychological problems
- Urinary tract infections (UTI) and dysuria

According to the members of the Delphi Panel, practically all the comorbidities and additional resource uses seen in VVA patients and related to this condition could be appropriately addressed with the existing available therapies.

On the other hand, the sites reported a marked additional unmet need in VVA patients with a history of BC, especially in the following areas:

- Overall treatment of VVA
- Urinary tract infections and dysuria
- Postmenopausal bleeding
- Reduced Quality of Life
- Gynecological visits

During the discussion and the follow-up interviews, UTIs were considered as clinically relevant problems because in untreated VVA patients, there is a tendency to recurrence.

The responses to this question confirm that ospemifene is perceived as an efficacious drug in all VVA patients.

There seems to be a tendency to privilege the use of ospemifene, at least as a first line, in VVA patients with a history of BC.

According to the Panel members, the main reason is the cost which – since the drug is not reimbursed in Italy – may be considered in some circumstances too high for patients who do not have a history of BC.

This is also an indirect confirmation of the fact that the overall situation of VVA patients with a history of BC is perceived as more severe and less manageable than in the normal population of these patients.

The additional burden of illness caused by VVA in patients was evaluated in a semiquantitative manner by the Panel members by comparing the results of a retrospective epidemiological, record-based study performed on 313,382 US patients (data on file, follow-up of a research published in [17]) with diagnosed VVA, with their clinical experience.

The study reported that the increase of the incidence (expressed in additional cases per 100 patients per year) found retrospectively for a series of events/comorbidities in patients with VVA, with or without a history of breast cancer.

The members of the Panel were asked to evaluate the plausibility of the causal relationship between the comorbidities/events reported in the US study and their clinical experience and to confirm or modify the expected number of additional cases reported in the US analysis.

The responses of the sites are summarized in Table 2.

**Table 2:** Estimated additional number of comorbidities/events (per 100 patients/year) associated with VVA by Delphi Panel components

Comorbidity/Event	VVA newly diagnosed, non-BC patients (additional cases/100 patients per year)	VVA newly diagnosed in patients with history of BC (additional cases/100 patients per year)
Urinary tract infections	3.59	4.25
Urinary frequency	1.3	1.96
Gyn. visits	32.67	33.4
Vulvovaginitis	3.46	3.68
Gen. tract symptoms	2.43	3.38
Cancer screening	17.59	18.48
Postmenopausal bleeding	0	3.13
Psych. Unadjustment	10	10
Climacteric symptoms	6.48	6.97
Bone and cart. disorders	4.03	3.64
Cystocele	1.6	1.81
Mastopathy	1.54	0
Outpatient visits	1.88	2.53
Stress incontinence	1.4	1.98
Screen for HPV	3.26	2.96

## Discussion

This study started from an extended set of epidemiological data originated in the US using a Health Claims database, a very accurate and reliable source of information.

Due to the lack of epidemiological evidence for Italy and to the difficulties of organizing an “ad hoc study,” a Delphi Panel seemed an adequate preliminary approach to assess the situation in a semi-quantitative way and to guide future initiatives.

The Delphi Panel, which inherently reduces group-related bias as much as possible, was used in fact to “bridge” the US data and to evaluate its applicability to the Italian reality.

Some of the conclusions from the US study, in particular those pertaining the urological and psychological implications of VVA, were confirmed by our Panel. Others, quite expectedly, were modified or rejected as not relevant to the local situation.

It can be stated, however, that the picture that emerged from the Panel adequately represents the impact of VVA in BC patients in Italy. The involved sites constitute a representative sample of the Italian patient population and of the diagnostic and treatment pathways applied in VVA patients with a history of BC.

The results of Delphi Panel were able to shed some light on the Italian situation and to confirm that also in Italy, VVA, especially in BC patients, is a socially relevant condition.

The results appear in line with several very recent papers that emphasize the importance of VVA in general [3, 4, 18–20] and in BC patients [21] and confirm that the condition still represents an unmet medical need [22].

Even though nothing can substitute an epidemiological prospective study, which will be needed to obtain a definitive and quantitative answer, the knowledge obtained through the Delphi Panel will be very useful in guiding the choices of such future study.

## Conclusions

The Panel essentially confirmed that VVA is a diffused and underdiagnosed condition.

In patients with a history of BC, signs and burden of illness of VVA tend to be more severe, as well as more difficult to manage due to the restricted number of therapeutic options [5, 23, 24].

Many of the conclusions of the US study on the comorbidities and events associated with VVA can be applied also to the Italian situation.

In particular, the Italian Panel agreed on the fact that genitourinary problems appear more frequent in a VVA population, including urinary tract infections, incontinence, and dysuria, all leading to a higher frequency of urological visits. Genitourinary problems, especially infections and dysuria, tend to be more severe in BC patients.

On the other hand, conditions reported in the US study, such as postmenopausal bleeding, hematuria, uterine leiomyoma and fibrocystic mastopathy were not considered so important VVA comorbidities in Italy.

The most relevant consequences of VVA are in the “uro-gynecological” and in the “psychological” domains, with a relevant impact on the life of the patients.

The Panel was in fact unanimous in confirming that depression and the need for psychological counseling are more frequent in VVA patients, along with difficulties in sex life and a reduced quality of life. Also these problems are more severe in VVA patients with a history of BC.

The diagnostic and therapeutic approach to VVA related conditions is consistent throughout all represented sites and involves also the use of a significant amount of NHS (National Health System) resources.

Given the limited therapeutic options available, BC survivors represent a relevant subpopulation of VVA patients that could benefit from new therapeutic approaches.

In particular, vaginal laser has shown a beneficial effect in treating VVA in BC survivors in the short term, but there is no sufficient data on the long-term treatment.

Moreover, a new drug-like ospemifene has demonstrated clinical safety on the breast tissue, and therefore it could be indicated in women with previous history of breast cancer who have completed treatment.

## Abbreviations

ACOG: American College of Obstetricians and Gynecologists
AIOM: Associazione Italiana di Oncologia Medica (Italian Association of Medical Oncology
AIRTUM: Associazione Italiana Registri Tumori (Italian Cancer Registry Association)
BC: Breast cancer
HPV: Human Papilloma Virus
NHS: National Health System
US: United States
UTI: Urinary tract infections
VVA: Vulvovaginal atrophy
